# Low Glycemic Index Carbohydrates versus All Types of Carbohydrates for Treating Diabetes in Pregnancy: A Randomized Clinical Trial to Evaluate the Effect of Glycemic Control

**DOI:** 10.1155/2012/296017

**Published:** 2012-11-29

**Authors:** Otilia Perichart-Perera, Margie Balas-Nakash, Ameyalli Rodríguez-Cano, Jennifer Legorreta-Legorreta, Adalberto Parra-Covarrubias, Felipe Vadillo-Ortega

**Affiliations:** ^1^Nutrition Research Department, Instituto Nacional de Perinatología Isidro Espinosa de los Reyes, Montes Urales 800, Lomas de Virreyes, 11000 Mexico City, Mexico; ^2^Endocrinology Department, Instituto Nacional de Perinatología Isidro Espinosa de los Reyes, Montes Urales 800, Lomas de Virreyes, 11000 Mexico City, Mexico; ^3^Unidad de Vinculación, Facultad de Medicina, UNAM, Instituto Nacional de Medicina Genomica, 04510 Mexico City, Mexico

## Abstract

*Background*. Due to the higher prevalence of obesity and diabetes mellitus (DM), more pregnant women complicated with diabetes are in need of clinical care. *Purpose*. Compare the effect of including only low glycemic index (GI) carbohydrates (CHO) against all types of CHO on maternal glycemic control and on the maternal and newborn's nutritional status of women with type 2 DM and gestational diabetes mellitus (GDM). *Methods*. Women (*n* = 107, ≤29 weeks of gestation) were randomly assigned to one of two nutrition intervention groups: moderate energy and CHO restriction (Group 1: all types of CHO, Group 2: low GI foods). *Results*. No baseline differences in clinical data were observed. Capillary glucose concentrations throughout pregnancy were similar between groups. Fewer women in Group 2 exceeded weight gain recommendations. Higher risk of prematurity was observed in women in Group 2. No differences in glycemic control were observed between women with type 2 DM and those with GDM. *Conclusions. *Inclusion of low GI CHO as part of a comprehensive nutrition intervention is equally effective in improving glycemic control as compared to all types of CHO. This strategy had a positive effect in preventing excessive maternal weight gain but increased the risk of prematurity.

## 1. Introduction

Diabetes mellitus (DM) is one of the leading causes of death among women in developing countries [1]. In Mexico, the prevalence of type 2 DM has increased markedly [2], in parallel with a 50% increase prevalence of overweight and obesity [3]. The latter represents a major risk factor for increasing the risk of type 2 DM and gestational diabetes (GDM) [4], associated with maternal and perinatal adverse outcomes [5, 6], and long-term chronic diseases [7]. GDM prevalence in Mexico varies between 8–12% [8, 9].

Maternal, fetal, and neonatal adverse outcomes can be significantly reduced when blood glucose levels are maintained within normal ranges throughout pregnancy with medical and nutritional treatment [10]. Intensive diet therapy is recommended to achieve optimal glucose control, to meet the energy and nutrients needs during pregnancy, and to promote adequate weight gain. Medical nutrition therapy is recommended for all pregnant women with DM [10, 11]. Carbohydrate (CHO) restriction, (40–45% of total energy intake (TEI)), moderate energy restriction (33%), and capillary glucose self-monitoring appear to be essential for achieving glycemic control in these women [12, 13]. Nutrition interventions with dietary advice as part of the management have improved maternal and perinatal adverse outcomes, when compared to routine care [14, 15]. However, at present, it is not clear if different types of CHO in the diet may exert different effects; although decreasing the glycemic index (GI) of the diet appears to have an additional positive effect on glycemic control in patients with DM [16]. In a systematic review, a low GI diet in noncomplicated pregnant women decreased the birthweight and the frequency of large- for gestational-age (LGA) newborns, when compared to a higher GI diet [17]. A recent study in women with GDM showed a decrease in insulin needs when a low GI diet was prescribed compared to the American Diabetes Association diet [18]. No studies have described the effect of a low GI diet on glycemic control in women with GDM or previous DM. The aim of this study was to compare the effect of including only low GI CHO in the diet against all types of CHO on maternal glycemic control and on the maternal and newborn's nutritional status of women with type 2 DM and GDM.

## 2. Materials and Methods

This paper reports the results of the two groups of pregnant women with DM who received a nutrition intervention within a randomized clinical trial (no. NCT00860613) conducted between 2004 and 2008. The Institutional Review Board of Instituto Nacional de Perinatología (Mexico City) approved the study protocol and the study was conducted according to the Declaration of Helsinki (as amended, October 2000).

Women were included if they had a gestational age ≤29 weeks, had GDM or pregestational type 2 DM, and planned to attend their pregnancy in our institution. At that time, universal 2 step screening for GDM at ≥14 weeks of gestation was done to all women. If the initial 50 g-1 hour (h) glucose challenge test was ≥130 mg/dL (7.2 mmol/dL), a 100 g-3 h oral glucose tolerance test was performed within two weeks and GDM was diagnosed with two or more abnormal values (fasting ≥ 95 mg/dL, 1 h ≥ 180 mg/dL, 2 h ≥ 155 mg/dL, and 3 h ≥ 140 mg/dL) [11]. Women with type 2 DM were diagnosed with a fasting glucose >126 mg/dL in early pregnancy (<14 weeks) or a random glucose >200 mg/dL. If women were already treated for type 2 DM before pregnancy, a new diagnosis was not made. Women were excluded if they had type 1 DM, renal, hepatic, or other metabolic diseases, or if they were unable to follow the nutrition intervention. 

Women were enrolled by the staff of the Nutrition Department at the Endocrinology Outpatient Clinic. The protocol for recruitment included an initial visit in which suitability for randomization was evaluated, an invitation to participate in the study was done, and an informed consent was obtained. Women were randomly assigned (simple randomization) to the study groups (parallel design) by a clinical dietitian, using a random number list and sequentially numbered files. The dietitian was blinded to the allocation schedule.

### 2.1. Intervention

#### 2.1.1. Group 1 (All Types of CHO)

The intervention followed the American Dietetic Association nutrition practice guidelines for gestational diabetes [19]. Women received an individual food plan based on CHO restriction (40–45% of TEI), using a CHO counting strategy (basic level) [20]. Moderate energy restriction was recommended only for overweight and obese women (24 kcal/kg). Breakfast CHO intake was limited to 15–30 g, and adequate fiber intake was promoted (20–35 g/day). Women in this group were advised to choose any type of CHO, except added refined sugars.

Energy and CHO prescriptions were revised at every visit and changes were done according to weight gain and whether or not ketonuria was present. If ketones were present and weight gain was subnormal, energy prescription was increased (200 to 300 kcal/day). If weight gain was adequate, energy was not modified and carbohydrates were increased (no more than 45% of TEI). Fat intake recommendation was maintained (<40% of TEI), and protein recommendation adjustment was made accordingly (20–25% of TEI). 

#### 2.1.2. Group 2 (Low GI CHO)

Women in this group received the same intervention as women in Group 1, but were counseled to eliminate all moderate and high GI foods (GI > 55) [21]. Tropical fruits, refined breads, breakfast cereals, flour tortilla, white rice, refined cookies and pastries, potatoes, carrots, beets, and refined sugars were eliminated from their plan. Papaya was the only moderate GI fruit permitted because it is one of the most frequently consumed high-fiber foods in this population. Corn tortillas were included only when combined with beans, as well as corn flakes combined with milk, according to some evidence that the combination of these foods decreases their GI [22, 23].

Women in both groups received the same individual nutrition education at each visit, following a specific protocol designed for this study. Educational themes included the importance of healthy eating in DM, identification of CHO, exchange lists and CHO counting, identification of high and low GI foods, healthy fats, and importance of capillary glucose self monitoring, among others. During each visit, concepts were reinforced, doubts were clarified, and women's skills in the application of the information provided were assessed (management of CHO servings, sample menus revision, etc.) assuring that all themes were covered during the intervention. Specific materials were designed for the educational purpose.

All women performed capillary glucose self-monitoring before and 2 h after meals (6 times/day, 2 days/week). Women were instructed on the use of the glucose meter and the recording of their glucose values in a specific format which included: time of the day, insulin dose, food intake, and glucose values before and 2 h after meals. At each visit, women reviewed their glucose monitoring records with the dietitian, identifying glucose values out of range and relating them with food intake.

### 2.2. Clinical Followup

Women received the same routine obstetric prenatal care. Obstetricians, endocrinologists, and laboratory personnel were not aware of the study groups. No women were using antidiabetic therapy. The endocrinologist was responsible for prescribing human insulin (intermediate-acting and regular) twice a day plus prandial rapid-acting insulin analogs as needed to meet glucose goals. 

Participants were followed-up every two to three weeks until the end of pregnancy. Nutrition baseline visits lasted 45 minutes, and followup visits were 30 minutes long. During each visit, nutrition assessment was done and nutrition recommendations were made accordingly. 

Diet adherence was measured as the mean energy intake adequacy throughout the intervention and it was calculated based on the energy intake reported each visit and the energy intake recommendation from the previous visit (% of energy adequacy = energy intake/energy recommendation × 100). Adequate adherence was considered to be 85–115%. 

### 2.3. Primary Outcomes

#### 2.3.1. Maternal Glycemic Control

Fasting plasma glucose concentrations were measured every two weeks by a glucose oxidase method. At the beginning of the study, all women received a glucose meter (Medisense Optium, Abbott Laboratories, USA) and glucose blood strips (Medisense, Abbott Laboratories, USA) for pre- and 2 h postprandial self-monitoring. Glucose goals were ≤95 mg/dL (5.27 mmol/L) and ≤120 mg/dL (6.66 mmol/L) for fasting and 2 h postprandial glucose, respectively. Insulin doses were reported by women in all visits.

### 2.4. Secondary Outcomes

#### 2.4.1. Maternal Nutritional Status

Weight was measured with a calibrated digital scale (TANITA, Tokyo, Japan) to the nearest ±0.1 kg, while the subject was wearing light clothing and no shoes. Height was measured with a digital stadiometer (SECA, Germany) to the nearest ±0.1 cm. Pregestational weight was self-reported and was used to obtain the prepregnancy body mass index. Obesity classification was done according to the World Health Organization criteria [24]. Total weight gain was assessed according to the U.S.A. Institute of Medicine recommendations [25]. Excessive weight gain was defined as >120% of recommended weight.

Diet was measured at baseline and every month (every 2 sessions) by the dietitian using the multiple-pass 24 h recall method. Food replicas were used to help with portion estimation. Nutrient analysis was performed using the Food Processor software (version 8.0, ESHA), which includes Mexican foods. Missing foods were added to the database using Mexican Tables of Nutritional Value [26]. The GI of the diet was calculated by multiplying the grams of CHO of each food consumed by the GI of that food, using glucose as standard, and adding up to a final number; that number was divided by the total grams of CHO consumed [27].

Women were instructed to collect their first morning urine sample in each visit and bring it to the dietitian. Ketones were measured with Multistix 10 SG (Bayer Diagnostics, Germany).

#### 2.4.2. Newborn's Nutritional Status

Birthweight at birth was measured with a calibrated digital scale (TANITA 1582 Tokyo, Japan) to the nearest ±0.1 kg. Length was measured using an infantometer (SECA 207, Germany) to the nearest ±0.1 cm. Head circumference was measured with a flexible nonstretchable measuring tape (SECA 212, Germany).

Nutritional status was assessed in term newborns, using the WHO reference data [28]. Malnutrition was defined as −2 standard deviations (SD) (weight/length for wasting, length/age for stunting) and <10 percentile for head circumference/age. Newborns were classified as small for gestational age (SGA) (weight/age < 10 percentile), LGA (weight/age > 90 percentile), low birthweight (LBW) (<2500 g), or macrosomia (>4000 g). For preterm newborns, the Babson reference data was used (SGA when weight/age < 10 percentile, LGA > 90 percentile) [29]. 

#### 2.4.3. Adverse Clinical Outcomes

Preeclampsia was diagnosed according to our institution guidelines (blood pressure > 140/90 mmHg, and proteinuria ≥ 300 mg/24 hours). Prematurity was considered when gestational age at birth was <37 weeks. These outcomes together with intrauterine and neonatal death were recorded from the medical chart.

### 2.5. Statistical Analysis

Sample size was estimated in order to obtain a difference of 10 mg/dL (sd ± 20 mg/dL) in the decrease of glucose throughout the intervention, with an 80% power and an alpha of 0.05, resulting in a minimum of 32 women in each group. All women were included in the analysis. Women lost to followup were assumed to maintain the last observation value (last observation carried forward imputation data) [30].

Fasting plasma glucose, insulin doses, and food intake were analyzed in three observation periods: a baseline (T-1), a middle value (T-2), and a last visit value (T-3). T1 for capillary glucose measurements was obtained 2 weeks after-intervention. Ten women did not have any capillary glucose values; thus, sample size for the analysis of this variable was smaller (*n* = 97). 

Descriptive statistics and frequencies for all variables were performed. Mean differences (Student's *t*-test or Mann-Whitney *U*-test for nonnormal distribution) were analyzed to assess correct randomization. Differences in proportions were done using chi-square and Fisher's exact test. Subgroup analyses were performed by type of DM. Intra and inter-group differences were assessed using repeated measures ANOVA, including study group and type of DM as factors. Multiple logistic regression was performed with type of DM and study group as independent variables and excessive maternal weight gain as dependent variable. Multiple logistic regression models were done for evaluating risk of adverse clinical outcomes. Type of DM, study group, presence of infection during pregnancy, and insulin use during pregnancy were the independent variables and clinical adverse outcomes were the dependent variables. A *P* value ≤ 0.05 was considered statistically significant. Statistics were done with the Statistical Package for the Social Sciences (SPSS) software, version 16.0 (Chicago, IL).

## 3. Results

**Figure 1:**
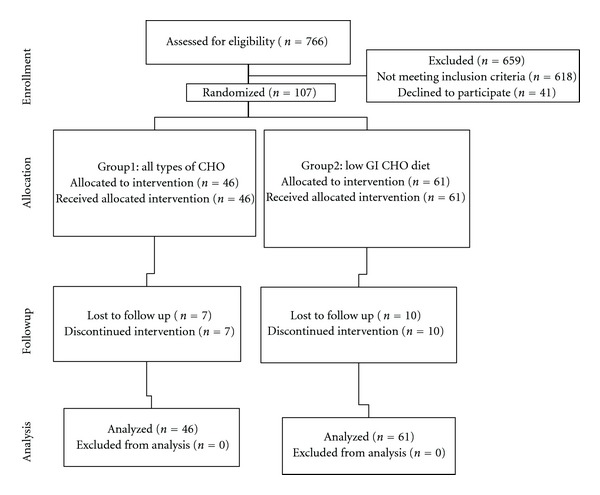
Flow diagram of trial.

We approached 766 women, but 618 did not meet the inclusion criteria and 41 refused to participate. From the total of women who met our eligibility criteria and agreed to participate (*n* = 107), 46 women were assigned to the low glycemic index dietary strategy and 61 women to the dietary strategy with all types of CHO (Figure 1). 

A total of 107 women received the nutrition intervention, 55 had type 2 DM and 52 had GDM. Mean age was 32 ± 5 years (range: 20–42 yrs) and mean gestational age at admission was 22 ± 6 weeks (range: 6 to 29 weeks). There were no baseline differences in personal, clinical, glycemic, anthropometric, and dietary data among the two groups (Tables 1 and 2). Pregestational overweight and obesity were present in 38.3% and 51.4% of women, respectively, without significant differences between groups. 

Clinical followup was similar between groups. Women in Group 1 had a mean of 7.09 ± 3.90 visits with the dietitian throughout the intervention period, and women in Group 2 had 5.93 ± 3.41 (*P* > 0.05). Overall, mean diet adherence was 83.05 ± 12.57% and was not different between groups (Group 1: 80.75 ± 11.62%, Group 2: 86.15 ± 18.08; *P* = 0.108). 

The proportion of women who discontinued the intervention (*n* = 17) was similar between the two groups (Group 1: *n* = 7, Group 2: *n* = 10). However, women were followed up until the end of pregnancy and were included analytically in the group to which they were randomized originally. 

### 3.1. Primary Outcomes

Fasting plasma glucose decreased significantly in Group 1 and Group 2 throughout the intervention (*P* < 0.003 and *P* < 0.004, resp.), but no significant differences were observed between groups. Capillary glucose concentrations throughout the intervention were similar among the two groups; the only significant decrease was observed in fasting capillary glucose (Table 3).

At the end of the intervention, the proportion of women who achieved glycemic goals at different mealtimes was similar between groups (*P* > 0.44). Women in Group 2 were successful in achieving glycemic goals at the end of pregnancy in 2 h postprandial glucose at lunch, preprandial, and 2 h postprandial glucose at dinner (*P* < 0.05). In Group 1, the only significant increase in the proportion of women meeting capillary glycemic goals was observed after lunch (*P* = 0.03) (Table 4).

Even though there was a high variability in the number of visits women had, no differences in fasting or capillary glucose throughout the intervention were observed between women who started the intervention earlier (<23 weeks of gestation) and women who started later. The proportion of women achieving glycemic goals at the end of pregnancy was also similar regardless of the number of visits (data not shown). 

The proportion of women who started using insulin during the intervention was similar between study groups in both types of DM (*P* ≥ 0.40), without differences in insulin doses (Group 1: 42.7 ± 26.4 u/d and Group 2: 35.5 ± 21.5 u/d) (*P* = 0.18). 

As expected, more women with type 2 DM used insulin during the intervention (90.9% versus 31.4%, *P* < 0.001) and with higher doses (41.02 ± 21.9 u/day versus 25.65 ± 19.36 u/day, *P* = 0.030) compared to women with GDM; however, no differences were observed by study groups in either type of DM. 

### 3.2. Secondary Outcomes

Women with type 2 DM gained more weight during pregnancy (7.5 ± 7.41 kg) when compared to women with GDM (4.35 ± 6.59 kg) (*P* = 0.023); no differences were observed by study groups. Regardless of the type of DM, a lower proportion of women in Group 2 (9.8%, *n* = 6) was classified as having excessive weight gain compared to women in Group 1 (34.8%, *n* = 16) ( *P* = 0.002) (RR: 3.53, 95% CI 1.50 to 8.32).

Energy and macronutrient intakes throughout pregnancy are described in Table 5. There was a significant decrease in the GI of the diet throughout the intervention period only in Group 2 (51.29 ± 7.28 to 47.18 ± 6.93, *P* = 0.001). No other significant dietary differences were observed between study groups. 

A trend for lower birthweight among newborns in Group 2 was observed (2883.9 ± 676.8 g versus 3115.5 ± 534.8 g) (*P* = 0.06) (95% CI of the difference: −13.55 to 476.72). The rate of macrosomia (Group 1: 6.8% versus Group 2: 3.4%, *P* = 0.649) and low birthweight (Group 1: 18.6% versus Group 2: 9.1%, *P* = 0.140) were similar between study groups. No inter-group differences in the frequency of SGA (Group 1: 6.8% versus Group 2: 10.2%) or LGA (Group 1: 4.5% versus Group 2: 5.1%) were detected. Wasting was present in 7.7% (Group 1) and 6.5% (Group 2) and stunting was observed in 4.5% (Group 1) and 11.9% (Group 2), without showing significant inter-group differences. No newborns from Group 1 had low head circumference, while 1 newborn in Group 2 was classified as having low head circumference (*P* ≥ 0.05). No differences by the type of DM were observed in any newborn nutritional marker.

No differences were observed between groups in the frequency of the clinical adverse outcomes studied (*P* > 0.05), but a trend of a higher frequency of prematurity was observed in Group 2 (19% versus 11.3%, *P* = 0.237). No differences were found in these clinical outcomes between women with GDM and type 2 DM within each study group (*P* > 0.05).

Multivariate analysis showed the same risk of preeclampsia, intrauterine, and neonatal death in women from both study groups, but a higher risk of prematurity in women in Group 2 (RR: 4.74, 95% CI: 1.08 to 20.84, *P* = 0.03). The model was adjusted by type of DM, insulin use, and presence of infection during pregnancy. 

## 4. Discussion

Nutrition intervention has been recognized as the cornerstone treatment for achieving glycemic control in pregnant women with DM [11, 19], and the general dietary guidelines for GDM treatment include CHO restriction and the promotion of adequate fiber and healthy fat intake [19]. No previous evidence exists regarding the effect of lowering the GI of the diet on glycemic control in pregnant women with type 2 DM or GDM. This study shows that including only low GI CHO in the diet is associated with similar glucose concentrations throughout pregnancy, when compared to women following the same energy and macronutrient prescription (40–45% of CHO, 20–25 g of fiber), but with moderate and high GI foods. However, as previously reported [11, 31, 32], the nutrition intervention had a positive effect in decreasing fasting plasma and capillary glucose throughout pregnancy in both groups and both types of diabetes. The proportion of women who achieved glycemic goals at the end of the intervention also increased at some mealtimes. Even though, there were no inter-group differences in capillary glucose, women in Group 2 achieved glycemic control at more mealtimes than women in Group 1 (intra-group comparisons). These findings are clinically important considering that the main goal of DM treatment in pregnancy is to achieve a consistent glycemic control throughout the day, and achieving postprandial glycemic control has been identified as a main factor for reducing DM-related complications [33].

A main strength of this study is that it provides data about the effect of low GI CHO on glycemic control and shows that this effect is not different between women with GDM and women with type 2 DM.

No differences in insulin users or doses were observed by study groups. In a recent study, fewer women with GDM, who were receiving a low GI diet, needed insulin during pregnancy, when compared to the American Diabetes Association diet [18]. Our results are not comparable, because in that study, a strict protocol to start women on insulin was followed. Insulin prescription was based on inadequate glycemic control and depended on the clinical judgment of the endocrinologist. 

Fewer women in the low GI group gained excessive weight, according to IOM guidelines [25]. This could be relevant because most women in our study were overweight or obese (90%), and it is well known that prepregnancy obesity and excessive gestational weight gain may increase the frequency of adverse pregnancy outcomes (preeclampsia, cesarean delivery, LGA, macrosomia, and/or neonatal adiposity) [34, 35]. 

Similar to a previous report [36], a trend to lower but within normal birthweight was observed in newborns of women in the low GI diet group, without increasing the frequency of SGA or LBW. Birthweight and weight indices have been used as indirect markers of intrauterine nutrition and fetal programming [37, 38] and the promotion of a healthy birthweight may be an important goal when treating obesity and DM in pregnancy. However, the implications that these findings may have in decreasing the short- and long-term nutritional and metabolic risks of the newborn are unknown.

A relevant finding was the higher risk of prematurity observed in women who followed the low GI dietary recommendations. This observation has not been reported previously, but there is evidence showing that dietary restriction during pregnancy is associated with higher risk of prematurity (OR: 1.14, 95% CI: 1.03 a 1.25, *P* = 0.009) [39]. The restriction of moderate and high GI foods in Group 2 may have resulted in a more restricted diet that could be associated with prematurity as well as with the observed lower frequency of excessive weight gain and the trend of lower birthweight in women in this group. However, our dietary data does not show any differences in energy intake between groups. 

Our study has some limitations that should be discussed. Firstly, bias may have been introduced by the fact that capillary glucose concentrations were self-recorded because of the lack of memory capacity of the glucose meters used [40]. Besides, baseline capillary glucose values were not available, since women received dietary and self-monitoring recommendations during the first prenatal visit, so the first capillary glucose value was already a result of the dietary intervention. It is likely that at the beginning of the study period (from the first to the second visit) a drop in capillary glucose values may have occurred, masking the expected intra-group decrease throughout pregnancy with nutrition interventions. In addition, insulin data was obtained from the medical prescription, and we assumed that women applied their insulin dose as prescribed. Secondly, a systematic error was introduced in the calculation of the diet GI due to the lack of GI data of some Mexican foods and food combinations. Finally, while the 24-hour-recall is useful in indirectly evaluating adherence to the nutrition intervention, the method has some limitations, including relying on the patient's memory, not evaluating usual energy and macronutrient intake, and the possibility of falsely reporting compliance with the intervention guidelines during an ongoing nutrition education process [41].

In conclusion, the inclusion of low GI CHO as part of a comprehensive nutrition intervention was equally effective in improving glycemic control as compared to all types of CHO, without differences between pregnant women with type 2 DM and women with GDM. The low GI strategy had a positive effect in preventing excessive maternal weight gain, but women in this group had higher risk of preterm birth. More data is needed before using the recommendation of restricting high and moderate GI foods for treating DM in pregnancy as a better strategy than including all types of CHO. Future research should evaluate the effects of a low GI diet on the risk of later developing type 2 DM in women with GDM and on newborn and child adiposity and metabolic markers.

## Figures and Tables

**Table 1:** Baseline personal, clinical, and glycemic data of pregnant women studied.

*n* = 107	Group 1 (All types of CHO) (*n* = 46)	Group 2 (Low GI CHO) (*n* = 61)	*P* value
Age (years)^a^	31.80 ± 5.3	32.30 ± 4.8	0.517
Type 2 DM (%, *n*)^b^	50.0 (23)	52.50 (32)	0.856
GDM (%, *n*)^b^	50.0 (23)	47.50 (29)	0.789
Gestational age at admission (weeks)^a^	20.70 ± 6.7	22.50 ± 4.9	0.182
Plasma fasting glucose (mg/dL)^c^	104.10 ± 31.83	95.02 ± 13.97	0.406
Capillary fasting glucose (mg/dL)^c^	96.00 ± 13.91	96.25 ± 12.21	0.531
Capillary 2 h postprandial glucose (breakfast) (mg/dL)^a^	105.45 ± 19.50	108.76 ± 19.10	0.404
Capillary 2 h postprandial glucose (lunch) (mg/dL)^a^	119.16 ± 16.15	118.79 ± 21.98	0.927
Capillary 2 h postprandial glucose (dinner) (mg/dL)^a^	108.97 ± 12.78	116.20 ± 21.96	0.503
Insulin users (%, *n*)^b^	69.60 (32)	56.70 (34)	0.192
Total insulin dose (units/day)^c^	29.10 ± 27.6	19.80 ± 22.5	0.078
Years diagnosed with type 2 DM (years)^c, d^	3.75 + 2.33	3.71 + 2.49	0.870
Oral hypoglycemic agents used before pregnancy (%, *n*)^b, d^	81 (17)	85.7 (24)	0.905

^a^Mean ± SD analyzed by Student *t*-test.

^b^Percentage (*n*) analyzed by Chi square test.

^c^Mean ± SD analyzed by Mann-Whitney *U* test.

^d^Women with type 2 DM (*n* = 49).

**Table 2:** Baseline anthropometric and dietary data of pregnant women studied.

*n* = 107	Group 1 (All types of CHO) (*n* = 46)	Group 2 (Low GI CHO) (*n* = 61)	*P* value
Weight (kg)^a^	73.95 ± 16.59	74.12 ± 13.72	0.953
Height (cm)^a^	152.36 ± 6.92	155.28 ± 5.17	0.054
Pregestational body mass index (kg/m^2^)^a^	32.0 ± 6.3	30.50 ± 5.2	0.132
Overweight/obesity (%, *n*)^b^	93.5 (43)	86.8 (53)	0.595
Energy intake (kcal/day)^c^	1525 ± 479	1535 ± 560	0.937
Carbohydrate intake (% TEI)^c^	47.50 ± 8.9	50.50 ± 8.6	0.418
Protein intake (% TEI)^a^	20.59 ± 4.91	19.59 ± 5.26	0.326
Lipid intake (% TEI)^a^	32.09 ± 8.41	29.80 ± 8.30	0.166
Fiber intake (g/day)^a^	23.10 ± 10.8	24.30 ± 12.8	0.804
Saturated fat intake (% TEI)^a^	10.00 ± 3.20	9.47 ± 3.49	0.433
Monounsaturated fat intake (% TEI)^a^	9.85 ± 4.31	8.46 ± 3.19	0.060
Polyunsaturated fat intake (% TEI)^c^	6.61 ± 4.26	5.17 ± 3.25	0.067
Glycemic index of diet^a^	50.0 ± 8.9	51.20 ± 7.2	0.712

^a^Mean ± SD analyzed by Student *t*-test.

^b^Percentage (*n*) analyzed by Chi square test.

^c^Mean ± SD analyzed by Mann-Whitney *U* test.

%TEI: total energy intake.

**Table 3:** Group differences in plasma and capillary glucose concentration (mg/dL) throughout the intervention (*n* = 97).

	Group 1 (All types of CHO) (*n* = 42)				Group 2 (Low GI CHO) (*n* = 55)				*P* value^b, c^
	T1	T2	T3	*P* value^a^	T1	T2	T3	*P* value^a^	
Plasma fasting (mg/dL)	104.10 ± 31.83	92.07 ± 17.05	90.06 ± 15.64	0.003*	95.05 ± 13.97	91.82 ± 12.39	88.18 ± 13.83	0.004*	0.502
Capillary (mg/dL)									
Fasting	96.00 ± 13.91	90.32 ± 11.25	85.57 ± 11.78	0.001*	96.25 ± 12.21	91.78 ± 8.40	88.20 ± 12.06	0.001*	0.375
2 h-postprandial breakfast	105.45 ± 19.50	108.43 ± 15.31	106.21 ± 17.00	0.817	108.76 ± 19.10	106.65 ± 12.35	103.89 ± 16.69	0.068	0.874
Preprandial lunch	90.88 ± 16.02	87.98 ± 12.17	88.92 ± 13.78	0.481	88.85 ± 11.79	87.95 ± 8.09	89.20 ± 12.20	0.868	0.074
2 h-postprandial lunch	119.16 ± 16.15	118.27 ± 14.96	115.59 ± 14.96	0.179	118.79 ± 21.98	114.35 ± 13.97	115.51 ± 17.82	0.344	0.351
Preprandial dinner	96.92 ± 13.37	98.46 ± 15.88	96.42 ± 16.70	0.849	99.18 ± 18.26	96.14 ± 9.38	96.22 ± 12.74	0.221	0.298
2 h-postprandial dinner	108.97 ± 12.78	111.75 ± 13.21	111.78 ± 15.42	0.346	116.20 ± 21.96	111.82 ± 13.79	110.37 ± 14.92	0.084	0.448

Mean ± SD analyzed by repeated measures ANOVA (factors: study group and type of DM).

^a^Intra-group differences, ^b^inter-group differences.

^c^Estimated effect size Eta^2^ *P* > 0.05 in all comparisons.

*Significant decrease throughout time (*P* < 0.05).

Type of DM was a nonsignificant factor in the analysis (*P* > 0.05).

T1: first visit value, T2: mean values throughout pregnancy, and T3: last visit value.

**Table 4:** Proportion of women who achieved glycemic goals in capillary blood before and after the intervention in both groups.

*n* = 97	Group 1 (All types of CHO) (*n* = 42)			Group 2 (Low GI CHO) (*n* = 55)		
	Baseline (%)	Final (%)	*P* value^a^	Baseline (%)	Final (%)	*P* value^a^
Fasting (mg/dL)	59.5	90.5	0.139	49.1	81.8	0.078
2 h-postprandial breakfast (mg/dL)	78.6	88.1	0.281	83.6	92.7	0.121
Preprandial lunch (mg/dL)	61.9	77.8	0.063	79.6	80.6	0.693
2 h-postprandial lunch (mg/dL)	52.4	61.9	**0.031**	64.8	70.4	**0.035**
Preprandial dinner (mg/dL)	42.9	57.1	0.087	50.0	57.4	**0.001**
2 h-postprandial dinner (mg/dL)	78.6	83.3	0.614	66.7	77.8	**0.037**

^a^Within-group difference in the proportion of women analyzed with chi-square test.

**Table 5:** Dietary intake throughout the intervention in both study groups.

*n* = 107	Group 1 (All types of CHO) (*n* = 46)				Group 2 (Low GI CHO) (*n* = 61)				*P* value^b, c^
	T1	T2	T3	*P* value^a^	T1	T2	T3	*P* value^a^
Energy (kcal)	1525 ± 479	1568 ± 478	1613 ± 474	0.299	1542 ± 566	1507 ± 379	1506 ± 438	0.697	0.838
Protein (% TEI)	20.58 ± 4.91	21.81 ± 4.79	21.15 ± 5.23	0.596	19.83 ± 5.45	23.54 ± 20.15	21.45 ± 4.42	0.058	0.582
Carbohydrates (% TEI)	47.50 ± 8.95	47.88 ± 6.84	45.78 ± 8.27	0.333	48.67 ± 8.61	47.59 ± 8.17	46.59 ± 9.09	0.957	0.530
Fiber (g/day)	23.11 ± 10.84	25.99 ± 11.55	20.58 ± 4.91	0.211	24.70 ± 12.84	26.81 ± 10.78	25.36 ± 10.31	0.632	0.666
Lipids (% TEI)	32.08 ± 8.41	30.26 ± 6.75	32.47 ± 7.36	0.800	29.45 ± 8.37	28.38 ± 9.96	27.90 ± 8.51	0.300	0.518
Saturated fat (% TEI)	10.00 ± 3.26	10.43 ± 3.20	10.60 ± 2.96	0.262	9.31 ± 3.56	8.51 ± 2.90	8.70 ± 3.27	0.326	0.473
Monounsaturated fat (% TEI)	9.84 ± 4.31	8.86 ± 2.74	8.86 ± 2.74	0.804	8.27 ± 3.29	8.31 ± 6.17	7.91 ± 3.23	0.521	0.114
Polyunsaturated fat (% TEI)	6.95 ± 3.97	5.06 ± 3.21	6.00 ± 4.11	0.190	5.49 ± 4.01	5.62 ± 4.96	4.96 ± 3.09	0.385	0.475
Glycemic index	50.00 ± 8.98	—	48.61 ± 8.37	0.345	51.29 ± 7.28	—	47.18 ± 6.93	0.001*	0.921

Mean ± SD analyzed by repeated measures ANOVA.

^a^Intra-group differences, ^b^inter-group differences.

^c^Estimated effect size Eta^2^ *P* > 0.05 in all comparisons.

*Significant decrease throughout time (*P* < 0.05).

T1: first visit value, T2: mean values during pregnancy, and T3: last visit value.

%TEI: Total energy intake.
